# Assessing the effect of a canine surgical-neutering educational programme on the knowledge and confidence of Indian veterinary participants

**DOI:** 10.3389/fvets.2023.942890

**Published:** 2023-05-25

**Authors:** Emma L. Rayner, Ilona Airikkala-Otter, Richard J. Mellanby, Andrew D. Gibson, Aswin Susheelan, Luke Gamble, Stella Mazeri

**Affiliations:** ^1^Worldwide Veterinary Service (WVS), Dorset, United Kingdom; ^2^Worldwide Veterinary Service India, International Training Centre, Nilgiris, Tamil Nadu, India; ^3^Royal (Dick) School of Veterinary Studies and the Roslin Institute, The University of Edinburgh, Easter Bush Campus, Midlothian, United Kingdom; ^4^The Epidemiology, Economics and Risk Assessment (EERA) Group, The Roslin Institute, Royal (Dick) School of Veterinary Studies, Easter Bush, Midlothian, United Kingdom

**Keywords:** veterinary education, India, canine surgical neutering, knowledge, attitudes, dog population management

## Abstract

India has a large, free-roaming dog population, encompassing both owned and stray dogs. Canine surgical neutering is often a central component of dog population management and rabies control initiatives. The provision of practical, surgical training opportunities remains a major challenge for veterinary educational establishments worldwide to ensure competency in this routine procedure. A 12-day educational programme, focusing on surgical neutering skills, was developed to address this need. A questionnaire comprising 26 questions covering surgical and clinical topics, and a self-assessment of confidence in undertaking five common surgical procedures, was completed immediately before and after finishing the programme. A total of 296 participants attended, with 228 achieving the inclusion criteria for the study. Total knowledge scores increased significantly after the training programme (mean score pre-18.94, 95% CI 18.13–19.74; post-28.11, 95% CI 27.44–28.77, *p* < 0.05) with improvements seen in all categories (surgical principles, anaesthesia, antibiotic use and wound management). After accounting for other participants’ characteristics, scores increased, on average, by 9 points after training. Being female was associated with significantly higher overall scores, while compared to younger and older age groups, those aged 25–34 were associated with lower overall scores. Amongst those with post-graduate qualifications, overall scores increased with age. Furthermore, there was an increase in self-rated confidence by participants in undertaking all five procedures. This study demonstrates that a targeted training programme can improve veterinary participants’ knowledge and confidence in canine surgical neutering and may provide an effective way to develop surgical expertise amongst veterinarians engaged in dog population management initiatives.

## Introduction

Veterinarians play a critical role in upholding the health and wellbeing of animals in India. Of notable national importance is the large, free-roaming dog population which is focused primarily in urban areas and in close association with people. Effective management of this population, comprising owned and ownerless dogs, is paramount in addressing key, human and animal health concerns (1). The training veterinarians receive must equip them with the skills necessary to address these critical One-Health issues, such as the elimination of rabies through canine vaccination and effective dog population management (DPM). Veterinary undergraduates in India undertake a five and a half-year degree programme at one of 46 veterinary colleges, with approximately 2,400 graduates annually. The curriculum is diverse, covering 18 non-clinical and clinical subjects and includes domestic, livestock and wildlife species; new graduates are expected to be competent in a number of varied roles, such as veterinary public health, livestock production management, and clinical practices including farm and companion animals (2). Veterinary establishments globally face the challenge of providing “the ideal veterinary degree to suit the needs of modern society” (3) whilst ensuring the provision of key skills in areas of specific national importance. The provision of continuing professional development (CPD) enables further opportunities for graduates to acquire and/or maintain skills in their chosen field of work. Such opportunities often vary in availability, and their success depends on factors such as affordability and accessibility (4), and relevance to the target audience.

In countries where DPM is necessary, the Food and Agriculture Organisation has identified potential opportunities to develop training programmes for veterinarians, in collaboration with NGOs, which focus on high welfare, surgical, DPM methods (1). The UK-based Worldwide Veterinary Service (WVS), an international charity, has been running residential 12-day surgical training programmes at its international training centres (ITCs) in Malawi, Thailand and India since 2010, with the aim of sustainably improving clinical standards of humane dog population control. The programme comprises a large practical component, augmented by lecture-based teaching.

This study was designed to assess the effect of this educational training programme on both participants’ knowledge of, and their self-reported confidence in, undertaking surgical procedures. Previous reports have demonstrated the effectiveness of educational training initiatives in improving knowledge of veterinary and animal health-related topics of veterinarians in India (5) and USA (6, 7) and non-veterinarians in India (8), Tanzania (9), and Ethiopia (10). However, to the authors’ knowledge, there are no published data on the delivery of a practically focused, training opportunity for veterinarians in canine surgical neutering. The following research question was developed: was there an immediate effect on veterinarians’ knowledge of surgical neutering and confidence in carrying out surgical neutering procedures after attending such a training opportunity? The aim of this study was, therefore, to evaluate the effect of a targeted, surgical-neutering training programme on Indian veterinary participants’ knowledge and self-reported confidence using pre-and post-training questionnaires.

## Materials and methods

### Ethical considerations

The project was approved by the University of Edinburgh Royal (Dick) School of Veterinary Studies Human Subject Ethical Review Committee in August 2020 (HERC_568-20). The WVS surgery programme was approved by the Animal Welfare Board of India (AWBI), Government of India.

### Educational training programme

This study was conducted at two ITCs located in the Indian states of Tamil Nadu and Goa; these were established by the WVS charity in 2010 and 2016 respectively. They provide practical, surgical training in humane DPM to national and international veterinary professionals and undergraduates through regular, 12-day, training programmes taught in English. The structure and delivery of each course was consistent across both sites and participants participated in spay-neuter surgeries under direct supervision of experienced, nationally trained, Indian veterinary staff employed by WVS; initially, staff receive full training and support in course delivery before starting. The majority of each day was dedicated to practical, surgical experience. Initially, participants were assigned into pairs and took turns to perform a midline ovariohysterectomy or castration, the latter employing a pre-scrotal incisional approach, or to monitor general anaesthesia, all under direct supervision of the veterinary staff. Staff continuously assessed participants’ confidence and competence through observation and verbal communication and consequently adjusted the level of supervision they received as the course progressed. Each participant operated on at least one animal per day and, on average, a total of 16 dogs (1:1 gender ratio) over the 12-day programme. In addition, theoretical knowledge was improved by a series of daily lectures coving asepsis, anaesthesia, analgesia, responsible use of antibiotics, fluid therapy, trauma wound management, DPM, animal welfare and rabies. Lectures were followed by discussion opportunities. The responsible use of antibiotics and animal welfare lectures included case-based, interactive-learning sessions, encouraging problem-solving using ‘real-life’ scenarios. Additionally, there was a series of practical demonstrations covering common surgical and preparatory procedures such as suturing, preparing surgical packs, and talking through practical considerations and approaches to complications such as a bleeding ovarian pedicle and emergencies such as cardiopulmonary resuscitation.

### Questionnaire

Pre-and post-training questionnaires were designed to test participants’ knowledge and confidence levels and formed part of the routine evaluation and feedback process for the training programme. These were identical apart from the inclusion of demographic questions in the pre-training questionnaire. At the beginning of the pre-training questionnaire, consent to use the data anonymously for the purpose of the study was requested from individual participants. Participants were identified through two, unique identification numbers generated by the participant and assigned through a randomised barcode number, to enable data linkage of pre-and post-training questionnaires. Demographic data comprised age, gender, country of origin, number of years since graduating, professional and employment status, and whether a career break had been taken in the last 6 months. Knowledge questions were designed around the pre-defined, learning objectives, assessing depth and breadth of knowledge in key subject areas. A total of 26 questions covered the following topics: surgical principles (*n* = 11), anaesthesia (*n* = 7), antibiotic use (*n* = 5) and wound management (*n* = 3). Two questions required free-text responses and the remainder were multiple choice options with single or multiple correct answers.

Participants were then asked to rate their confidence in performing a bitch spay and a dog castration, locating and stopping bleeding from the ovarian pedicle, placing an intravenous cannula correctly and dealing with anaesthetic emergencies. Responses were chosen from an ordinal scale: 1-“not confident even with one-to-one direct supervision”; 2-“confident with a supervisor vet scrubbed in assisting”; 3- “confident with a supervisor vet observing”; 4-“confident with a supervisor vet in the room if needed”; and 5-“confident without any supervision available.” Both questionnaires were completed under examination conditions. Topics included for both the knowledge and the self-reported confidence assessments were covered during the training programme, either through practical opportunities or through lectures and discussions. Opportunities were provided for participants to discuss their results and obtain feedback on their performance with staff after completion of the post-training questionnaire. The questionnaires were piloted initially on WVS staff members and a small number of participants and the results evaluated. Feedback was obtained about factors such as clarity and ease of use to improve wording and content. The final questionnaires were then used routinely for each programme (see Supplementary material).

Data were collected in both ITCs between August 2020 and December 2021. This covered 44 training programmes in total with, on average, 5 participants per course at the ITC in Goa and 12 participants per course at ITC Tamil Nadu. Participants completed the pre-training questionnaire during induction (day 1), and the post-training questionnaire on the last day (day 12). All questions were pre-programmed into a mobile device app designed by the WVS charity for the purpose of offline data collection in field conditions (11), and this app was used to input the answers at the time of assessment. The data were then synchronised to a cloud-based server for review and analysis through a secure website login.

### Data analysis

Data were included from participants who specified their country of origin as India, and who completed both the pre-and post-training assessments. Incomplete assessments, or participants from other countries, were excluded.

### Questionnaire scoring system

For multiple choice questions requiring a single correct answer, a score of 1 was assigned for the correct response and 0 for an incorrect response. A score of 1 was also awarded for each correct response in the two free-text questions. For the remainder, comprising multiple choice questions with greater than one correct answer, a score of 1 was assigned for each correct response. For each incorrect response, a score of-1 was assigned. The maximum score possible was 40. To enable assessment of changes in confidence ratings, an improvement in confidence levels was re-categorised as follows: categories 4 and 5 were classed as “confident” and categories 1–3 were classed as “not confident.”

### Statistics

Data analysis was carried out using the R Statistical programme version 3.6.1 (12) within RStudio 2020 v1.3.1073 (13). R package ggplot2 (14) was used to generate graphs. The significance level was set at 0.05. An exact binomial test was used to estimate confidence intervals of proportions using R package stats (12). A paired *t*-test was used to compare scores before and after the educational intervention. A mixed effects multivariable linear regression model was built, using questionnaire overall score as the outcome variable and participant as a random effect. The aim of model was to estimate the difference between pre and post-training scores, adjusting for other factors which included age, gender, professional and employment status, number of years since graduation and whether a career break had been taken in the last 6 months. The model was fit using R package lme4 (15). Variable selection was carried out using manual backwards elimination and meaningful interaction terms were considered. These included age with educational status, years since graduation with educational status and age with years since graduation. The final model was selected based on lowest Akaike information criterion (AIC) and simplicity.

## Results

### Demographics

Between August 2020 and December 2021, a total of 296 participants completed the questionnaire; out of these, 228 matched the inclusion criteria and were included in the study. Not all participants who attended the course completed the questionnaire; common reasons included arriving late once the course commenced or being otherwise engaged when the questionnaires were completed.

Demographic data for participants included in the study are shown in Table 1.

**Table 1:** Demographic data for participants (*n* = 228).

Demographic data		Number (%)	Number missing
Age range (mean/median)		21–53 (28.03/26)	1
Gender	Male	140 (61.40)	
	Female	87 (38.16)	
	Rather not say	1 (0.44)	
Professional status			
Undergraduate		21 (9.21)	
Graduate	Employed	79 (34.65)	
	Unemployed	65 (28.51)	
	Studying for PG qualification	13 (5.70)	
Post-graduate	Employed/Unemployed	50 (21.93)	
Education status	Student	21 (9.21)	
	Graduate	144 (63.16)	
	Postgraduate	63 (27.63)	
Number of years since graduation*			186
	0–1	48 (35.56)	
	2–5	49 (36.30)	
	6–10	21 (15.56)	
	>10	17 (12.59)	
Returned from career break in last 6 months*			120
	No	107 (63.69)	
	Yes	27 (16.07)	
	Not applicable	34 (20.24)	

*Questions added during the study, so earlier questionnaires do not contain this information.

Participant age varied between 21 and 53 years. The majority were male (61.40%, 140/228), and were graduates (63.16%, 144/228) or post-graduates, i.e., having obtained a post-graduate qualification (27.63%, 63/228). Over 70% of all participants had graduated from their most recent qualification within the last 5 years. Amongst the graduates, 50.32% (79/157) were employed whilst 41.40% (65/157) were unemployed and 8.28% (13/157) were undertaking further studies. Out of those that responded, 63.69% (107/168) reported they had not taken a career break in the last 6 months, whilst 16.07 (27/168) had taken a break.

### Knowledge

**Figure 1:**
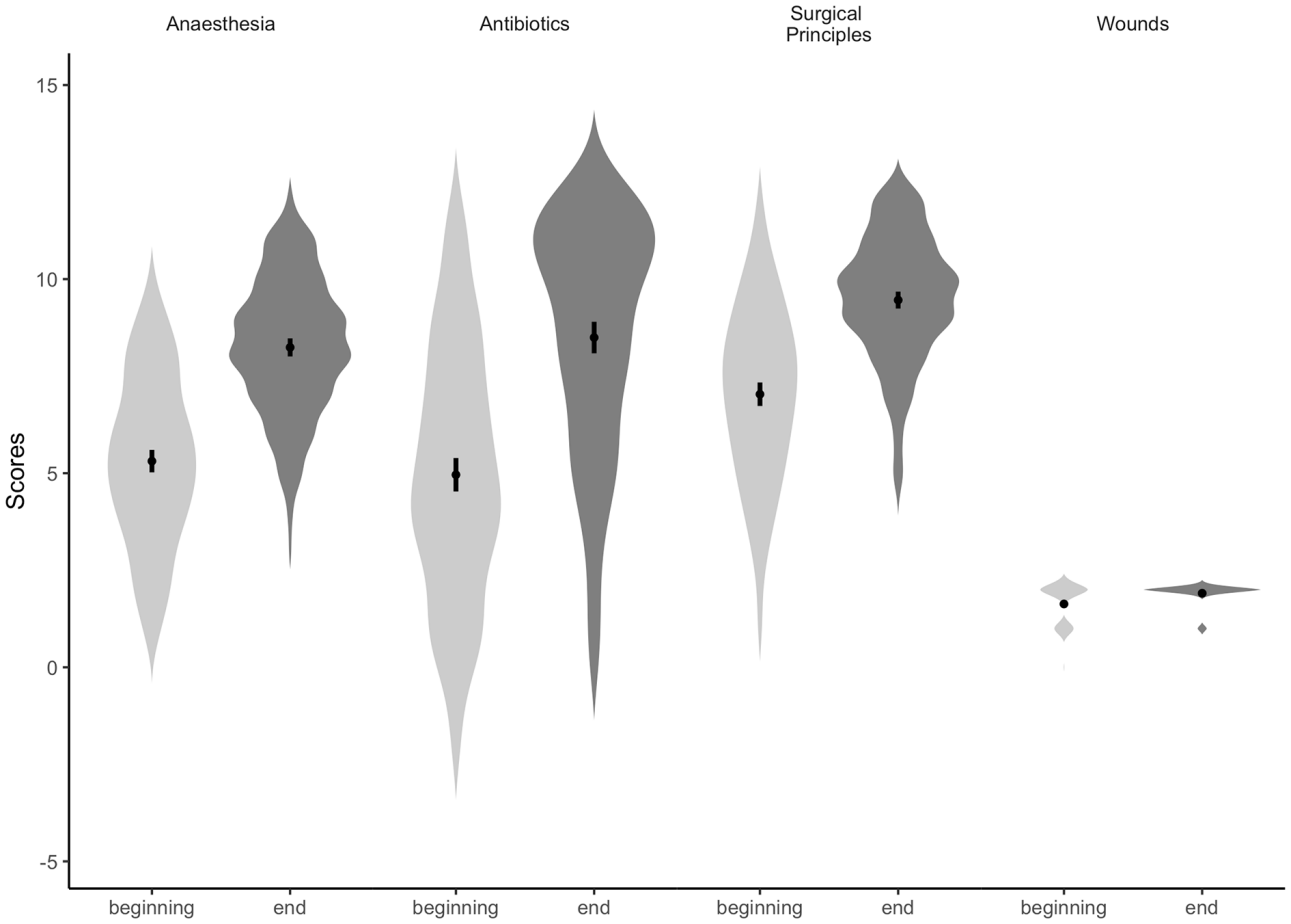
Violin plots to show the distribution of participants’ total scores for each knowledge category before and after the educational intervention.

**Figure 2:**
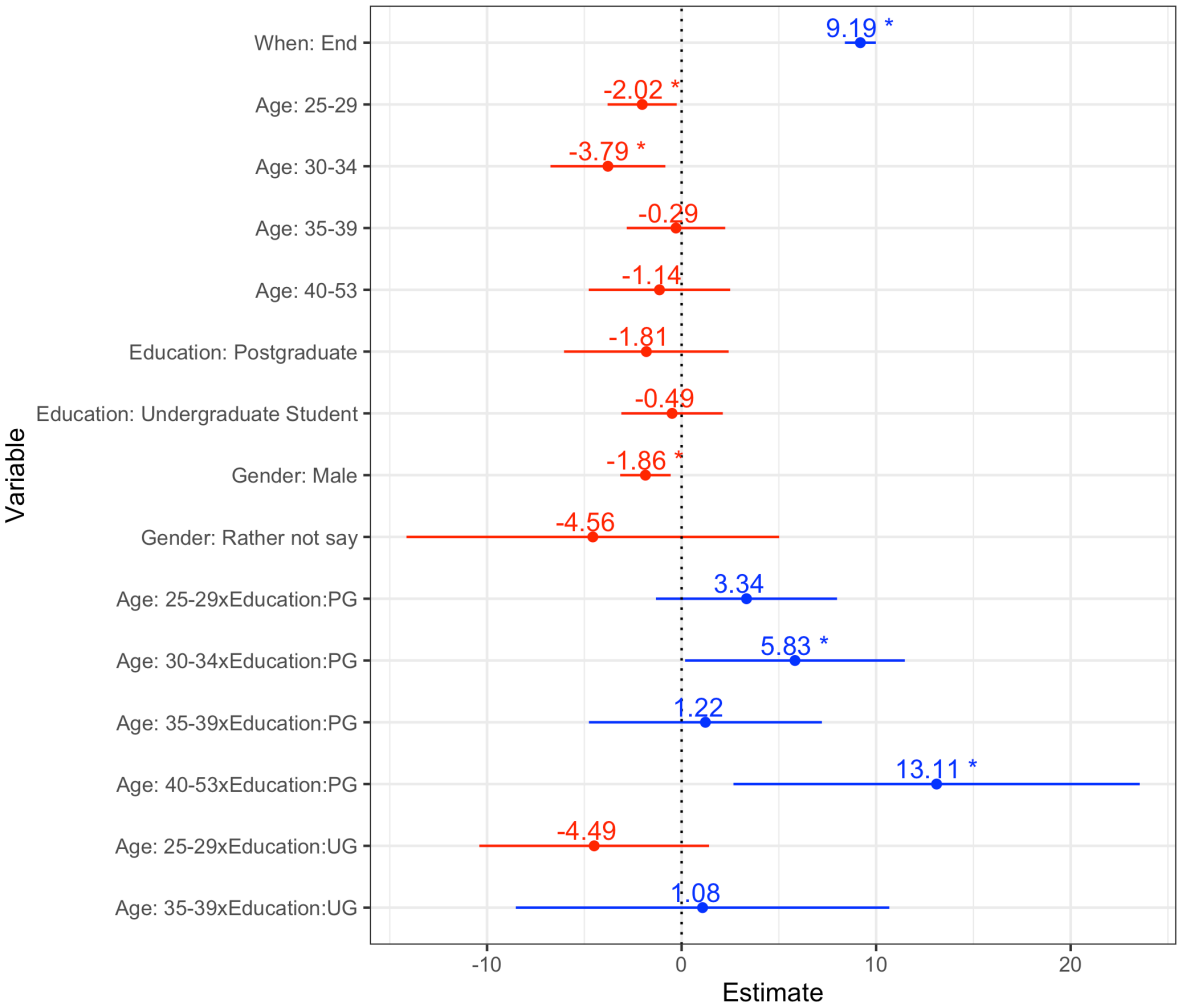
Multivariable linear regression model estimating differences in scores before and after training, adjusting for participants’ characteristics. * = <0.05, x = interaction.

The total knowledge scores of participants increased after attending the surgical training programme. The overall mean scores increased from 18.94 (18.13–19.74) to 28.11 (95% CI 27.44–28.77). Within individual categories, an increase in total scores was seen after the intervention as compared to before, the differences of which were statistically significant (paired *t*-test *p* < 0.05) (Figure 1). Values for individual categories are summarised in Table 2. Summary scores for each dependent variable can be found in the Supplementary materials. Results of the mixed effects linear regression model estimating the difference in scores before and after the training are shown in Figure 2. The final model indicated that average knowledge scores increased by 9.19 (95% CI 8.40 to 9.99) points at the end of the programme as compared to before, after accounting for other participants’ characteristics. Compared to males, females were associated with significantly higher scores. Compared to the youngest age group (21–24 years) and older age groups, those between 25 and 34 years old had significantly lower scores. Scores increased with age in those with post-graduate qualifications.

**Table 2:** Mean knowledge scores attained in each category before and after the training intervention.

Knowledge categories number of questions per category	Mean participant scores (95% CI)		
	Pre-training assessment	Post-training assessment	Paired *t*-test
Surgical principles (13)	7.04 (6.73–7.34)	9.46 (9.24–9.68)	<0.001
Anaesthesia and analgesia (12)	5.31 (5.02–5.60)	8.24 (8.01–8.47)	<0.001
Antibiotics (12)	4.96 (4.53–5.39)	8.50 (8.09–8.90)	<0.001
Wounds (3)	1.63 (1.56–1.70)	1.91 (1.87–1.95)	<0.001
Cumulative total (40)	18.94 (18.13–19.74)	28.11 (27.44–28.77)	<0.001

### Confidence

**Figure 3:**
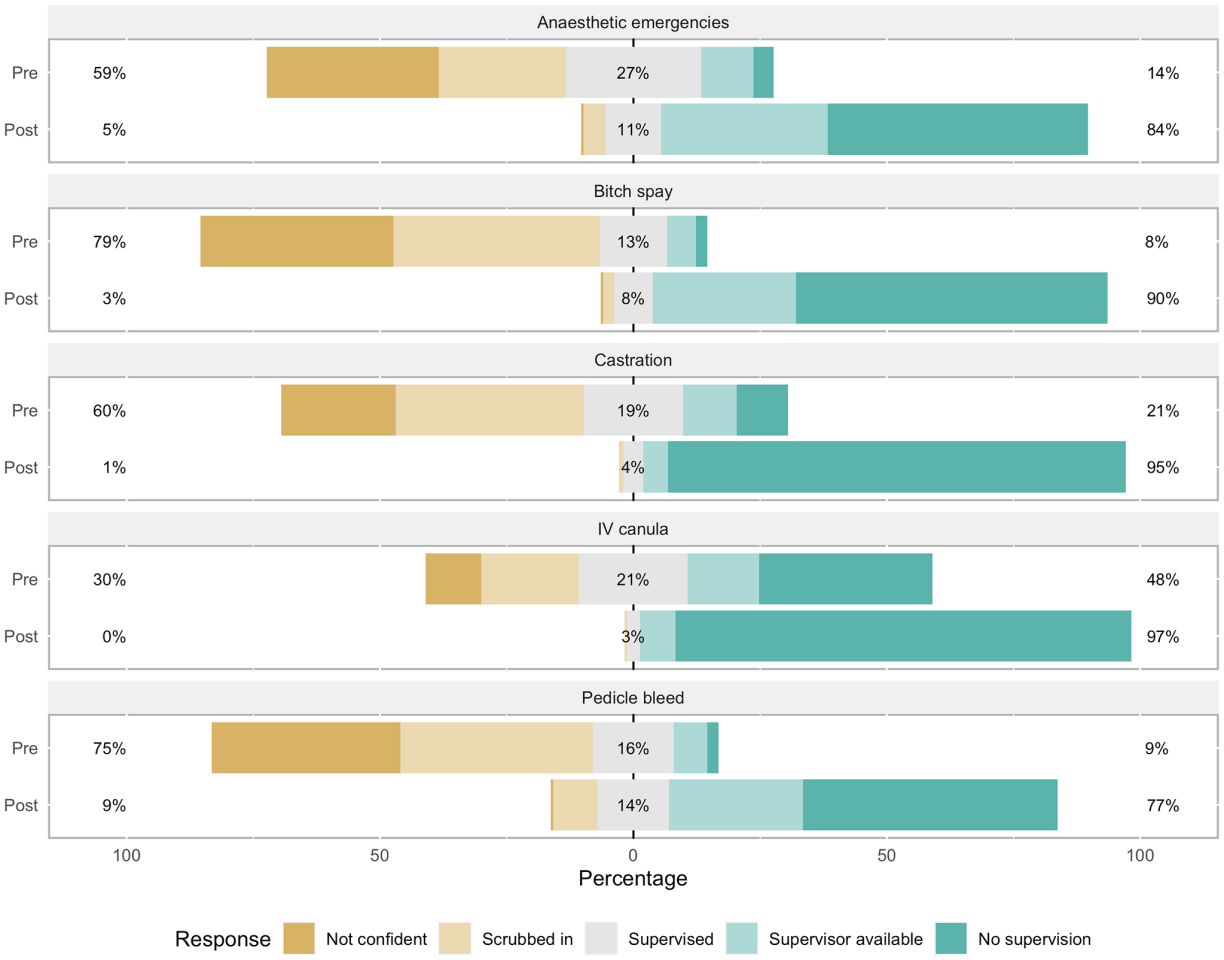
Responses of participants to questions relating to their confidence in undertaking key clinical and surgical procedures.

Upon completion, participants reported a notable increase in confidence in undertaking all five procedures, as compared to pre-training programme confidence levels (Figure 3). The percentage of participants classifying themselves as ‘confident’ (Scores 1 and 2) or not confident (Scores 3–5) pre-and post-training were compared. The percentage reporting they were confident in the various categories ranged between 8 and 48% prior to completing the programme; after completion, this increased to between 77 and 97%. Improvement in confidence, denoted by the difference in confidence scores before and after the training, was greatest for performing a bitch spay (82%), followed by performing a castration (74%), dealing with an anaesthetic emergency (70%) dealing with a haemorrhaging pedicle (68%), and placing an IV cannula (49%).

## Discussion

The results demonstrate that the level of knowledge and self-reported confidence of veterinary participants in canine surgical neutering improved significantly after attending the programme.

A significant increase in total knowledge scores was noted in participants after they had completed the educational training programme. The curriculum was delivered primarily in an approach that focused on active learning through practical experience, in an encouraging and supportive environment. This methodology followed the premise that ‘what the student does is more important in determining what is learned than what the teacher does’ (16). Practical teaching methods have become increasingly incorporated into veterinary curricula including the UK, United States and Australia due to their sound, evidence-based approach in providing students with the necessary skills to ensure competency, as compared to traditional, didactic methods (3). Our results support this style of training programme as an option for improving knowledge and practical skills, and the findings contribute to the overall evidence-based methodology focusing on the provision of educational initiatives to veterinarians in India. Furthermore, it highlights the capability of additional stakeholders, such as charities like the WVS, in achieving these goals.

DPM can help reduce human-animal conflicts and contributes to the maintenance of adequate vaccination coverage of zoonotic diseases, such as rabies, by reducing dog population turnover (17). The teaching material focused on the role of surgical neutering in DPM, which is under-represented in the current, local curriculum (2), with the aim of equipping veterinarians with key, surgical neutering skills and competency in running a successful spay-neuter programme.

A significant improvement in knowledge scores was seen in all, individual subject areas. These categories cover key skills necessary for veterinarians to ensure patient safety; included is the critical, global One Health focus on responsible antibiotic use. The need for greater awareness of antimicrobial resistance (AMR) amongst Indian veterinarians has been identified (18, 19). Furthermore, there is widespread, unregulated use of antibiotics amongst other national stakeholders, such as smallholder dairy farms (20). Opportunities for improved regulatory control of ‘over-the-counter’ sales of antibiotics in India have been identified to help address AMR (21). Improved veterinary working practices and client communication, achievable through focused training opportunities such as this, are also a key factor in combating this global threat to health.

Female participants were associated with significantly higher scores. The influence of gender on student academic performance has been reported previously in veterinary medical training (22) as well as wider academic subjects (23), with female students attaining higher grades. Furthermore, it may be hypothesised that younger participants, exposed more recently to the academic environment, were able to utilise the training more efficiently, building on their more recently updated, pre-training knowledge. These findings highlight the importance of ensuring a learning experience that demonstrates inclusion of a wide demographic of participant, thereby promoting equal opportunities and catering for all learning styles and career stages.

Participants reported that, when carrying out common procedures, their confidence levels were higher after attending the programme. The focus on ensuring sufficient opportunities to improve practical skills in surgery was adopted to address the commonly reported issue faced by veterinary graduates regarding sub-optimal confidence in surgical skills. An Australian study showed that only 75% of veterinary students had the opportunity to perform a canine ovariohysterectomy (OHE) surgery prior to graduation and yet 95% of graduates had to undertake this surgery without supervision soon after graduation (24). In a separate study, it was reported that canine OHE was the surgical procedure that 80.4% of final year, UK undergraduates were most concerned about regarding their ability to perform correctly (25). Newly qualified graduates with 6 months experience, reported a complication rate of 56.8% when performing canine OHE (26). Although no published data exists, it is likely that similar concerns are shared amongst veterinarians in India, particularly as ‘hands on’ experience was identified as a weakness in a SWOT (‘strengths, weaknesses, opportunities, and threats’) analysis of the veterinary curriculum (27).

There were a number of limitations in this study; firstly, identical pre-and post-training questionnaires were used to assess participants. For the knowledge assessment, participants may have guessed or assumed that the same questions would be asked at the end of the course and could have intentionally or unintentionally sought out answers during the course period. Evaluation of knowledge was limited to this method. For the self-assessment of confidence, a response bias may have affected participants’ scores. The study focused on the immediate effect of the training programme, thereby satisfying only level 1 of Kirkpatrick’s ‘Four Levels of Training Evaluation’ which is a globally recognised method of assessing learning programmes (28); however, future studies are proposed to examine whether participants’ behaviours changed in their workplace setting as a result of attendance to identify potential, longer-term impacts. Lastly, it is recognised that although the questionnaire design was tested prior to use, areas for improvement in question structuring could help when developing future studies.

Improvements in knowledge and confidence of veterinarians attending the training programme are encouraging and support the creation and delivery of future educational opportunities which build on the current training and experience of participants. Delivering the depth and breadth of curricula that includes sufficient development in surgical skills, is a universal challenge faced by both medical and veterinary teaching establishments. This challenge is amplified in low-resource settings where multiple barriers to success can exist; for example, substantial financial, geographical and cultural barriers, such as lack of training and equipment, and insufficient infrastructure, were identified in the delivery of human, surgical teaching programmes in resource-limited settings in 21 countries (29). In addition, many health-care interventions lack understanding of local issues, thereby failing in their delivery of vital outputs (29). Despite this, competency-based surgical training delivered in relatively short timeframes, and which can be incorporated into surgical outreach missions, have been shown to be successful in low and middle-income countries (30). In our study, we were able to demonstrate the effectiveness of a training programme delivered sustainably in a low-resource setting by local veterinary staff who understood local contexts, thereby overcoming potential barriers such as cultural sensitivities and equipment availability. WVS runs similar training programmes in countries such as Thailand, Malawi and the Galapagos islands, using the same model of long-term sustainability and locally employed workforce; therefore, the potential exists to expand these types of programmes through collaboration with local governmental organisations or teaching establishments to promote transfer of knowledge and build on local expertise in specific areas, such as DPM.

## Conclusion

Indian veterinarian participants’ knowledge of and confidence to undertake canine surgical neutering can be significantly increased through a targeted and practically focused, educational training programme, improving knowledge around key subject areas, and enhancing confidence in undertaking key skills required for canine surgical neutering. By demonstrating that a short, focused training programme has the capability to increase knowledge and confidence in a low-resource setting, this training programme provides an opportunity to build expertise amongst the veterinary community in India, as well as similar, global initiatives.

## Data availability statement

The raw data supporting the conclusions of this article will be made available by the authors, without undue reservation.

## Ethics statement

The studies involving human participants were reviewed and approved by the University of Edinburgh Royal (Dick) School of Veterinary Studies Human Subject Ethical Review Committee in August 2020 (HERC_568-20). The patients/participants provided their written informed consent to participate in this study.

## Author contributions

ER and IA-O played a major role in the conception and design of the study, with input also from AG, RM, SM, AS, and LG. AS was involved in overseeing the data collection. SM was involved primarily in the data analysis and focus of the results alongside ER. ER wrote the manuscript with contributions from all authors.

## Funding

SM was supported by Biotechnology and Biological Sciences Research Council through the Institute Strategic Program funding (BB/J004235/1 and BB/P013740/1).

## Conflict of interest

The authors declare that the research was conducted in the absence of any commercial or financial relationships that could be construed as a potential conflict of interest.

## Publisher’s note

All claims expressed in this article are solely those of the authors and do not necessarily represent those of their affiliated organizations, or those of the publisher, the editors and the reviewers. Any product that may be evaluated in this article, or claim that may be made by its manufacturer, is not guaranteed or endorsed by the publisher.
