# Supplementary material for: Assessing the effect of a canine surgical-neutering educational programme on the knowledge and confidence of Indian veterinary participants
Source: Front Vet Sci. 2023 May 25;10:942890. doi: 10.3389/fvets.2023.942890 (PMC10249432; doi:10.3389/fvets.2023.942890)
Supplement: Supplementary file 1 [file Table_1.DOCX]

Supplementary data

Tables to summarise the results of the multivariable linear regression modelling of knowledge scores.

Table S1: Multivariable regression model of knowledge scores results

| **Variable** | **Estimate** | **SE** | **95% CI** | **P value** |
| --- | --- | --- | --- | --- |
| When: End | 9.189 | 0.405 | 8.394 to 9.985 | 0.000 |
| Age: 25-29 | -2.025 | 0.905 | -3.803 to 9.985 | 0.026 |
| Age: 30-34 | -3.787 | 1.503 | -6.741 to -0.247 | 0.012 |
| Age: 35-39 | -0.289 | 1.288 | -2.821 to -0.833 | 0.823 |
| Age: 40-53 | -1.137 | 1.850 | -4.773 to 2.243 | 0.539 |
| Education: Post-graduate | -1.812 | 2.153 | -6.044 to 2.499 | 0.401 |
| Education: Undergraduate | -0.488 | 1.327 | -3.097 to 2.420 | 0.713 |
| Gender: Male | -1.859 | 0.660 | -3.156 to 2.120 | 0.005 |
| Gender: rather not say | -4.562 | 4.874 | -14.142 to -0.562 | 0.350 |
| Age: 25-29* Education: PG | 3.336 | 2.369 | -1.319 to 7.992 | 0.160 |
| Age: 30-34* Education: PG | 5.829 | 2.873 | 0.182 to 11.476 | 0.043 |
| Age: 35-39* Education: PG | 1.224 | 3.046 | -4.763 to 7.212 | 0.688 |
| Age: 40-53* Education: PG | 13.109 | 5.313 | 2.666 to 23.551 | 0.014 |
| Age: 25-29* Education: UG | -4.494 | 3.004 | -10.398 to 1.411 | 0.135 |
| Age: 30-34* Education: UG | 1.078 | 4.885 | -8.522 to 10.678 | 0.825 |

Table S2. Summary statistics of baseline knowledge scores by age category

|  | **Score** | | | |
| --- | --- | --- | --- | --- |
| **Age category** | **Minimum** | **Maximum** | **Mean** | **Median** |
| 20 - 24 | 3 | 30 | 19.72308 | 20.0 |
| 24 - 29 | 3 | 30 | 18.55769 | 19.5 |
| 29 - 34 | 7 | 33 | 18.58333 | 19.0 |
| 34 - 39 | 6 | 30 | 18.44000 | 19.0 |
| 39 - 53 | 9 | 32 | 19.33333 | 18.0 |

Table S3. Summary statistics of baseline knowledge scores at the beginning and end of training.

|  | **Score** | | | |
| --- | --- | --- | --- | --- |
| **Stage of course** | **Minimum** | **Maximum** | **Mean** | **Median** |
| Beginning | 3 | 33 | 18.93860 | 20 |
| End | 13 | 37 | 28.10526 | 29 |

Table S4. Summary statistics of baseline knowledge scores by gender.

|  | **Score** | | | |
| --- | --- | --- | --- | --- |
| **Gender** | **Minimum** | **Maximum** | **Mean** | **Median** |
| Female | 7 | 33 | 20.71264 | 21.0 |
| Male | 3 | 32 | 17.85714 | 17.5 |
| Rather not say | 16 | 16 | 16.00000 | 16.0 |

Table S5. Summary statistics of baseline knowledge scores by qualified versus student.

|  | **Score** | | | |
| --- | --- | --- | --- | --- |
| **Professional status** | **Minimum** | **Maximum** | **Mean** | **Median** |
| Qualified | 3 | 33 | 18.97585 | 20 |
| Student | 3 | 28 | 18.57143 | 20 |

Table S6. Summary statistics of baseline knowledge scores by education status.

|  | **Score** | | | |
| --- | --- | --- | --- | --- |
| **Education status** | **Minimum** | **Maximum** | **Mean** | **Median** |
| Graduate | 3 | 32 | 18.47917 | 18 |
| Post-graduate | 3 | 33 | 20.11111 | 21 |
| Undergraduate | 3 | 28 | 18.57143 | 20 |

Table S7. Summary statistics of baseline knowledge scores by years since graduation.

|  | **Score** | | | |
| --- | --- | --- | --- | --- |
| **Years since graduation** | **Minimum** | **Maximum** | **Mean** | **Median** |
| 0-1 | 3 | 27 | 18.64583 | 18 |
| 2-5 | 3 | 30 | 19.55102 | 21 |
| 6-10 | 7 | 33 | 19.90476 | 20 |
| >10 | 6 | 32 | 18.82353 | 18 |

Table S9. Summary statistics of baseline knowledge scores by career break

|  | **Score** | | | |
| --- | --- | --- | --- | --- |
| **Career break** | **Minimum** | **Maximum** | **Mean** | **Median** |
| Break | 9 | 30 | 17.48148 | 16 |
| No break | 3 | 32 | 18.36449 | 18 |
| Not applicable | 3 | 33 | 19.17647 | 20 |
